# Obesity-Linked Cancers: Current Knowledge, Challenges and Limitations in Mechanistic Studies and Rodent Models

**DOI:** 10.3390/cancers10120523

**Published:** 2018-12-18

**Authors:** Yang Xin Zi Xu, Suresh Mishra

**Affiliations:** 1 Department of Physiology and Pathophysiology, Rady Faculty of Health Sciences, Max Rady College of Medicine, University of Manitoba, Winnipeg, MB R3E 3P4, Canada; xuyxzc@myumanitoba.ca; 2 Department of Internal Medicine, Rady Faculty of Health Sciences, Max Rady College of Medicine, University of Manitoba, Winnipeg, MB R3E 3P4, Canada

**Keywords:** adipose tissue, insulin resistance, meta-inflammation, obesity-linked cancer models, hyperinsulinemia, adipose-immune crosstalk

## Abstract

The worldwide prevalence of obesity has doubled during the last 50 years, and according to the World Obesity Federation, one third of the people on Earth will be obese by the year 2025. Obesity is described as a chronic, relapsing and multifactorial disease that causes metabolic, biomechanical, and psychosocial health consequences. Growing evidence suggests that obesity is a risk factor for multiple cancer types and rivals smoking as the leading preventable cause for cancer incidence and mortality. The epidemic of obesity will likely generate a new wave of obesity-related cancers with high aggressiveness and shortened latency. Observational studies have shown that from cancer risk to disease prognosis, an individual with obesity is consistently ranked worse compared to their lean counterpart. Mechanistic studies identified similar sets of abnormalities under obesity that may lead to cancer development, including ectopic fat storage, altered adipokine profiles, hormone fluctuations and meta-inflammation, but could not explain how these common mechanisms produce over 13 different cancer types. A major hurdle in the mechanistic underpinning of obesity-related cancer is the lack of suitable pre-clinical models that spontaneously develop obesity-linked cancers like humans. Current approaches and animal models fall short when discerning the confounders that often coexist in obesity. In this mini-review, we will briefly survey advances in the different obesity-linked cancers and discuss the challenges and limitations in the rodent models employed to study their relationship. We will also provide our perspectives on the future of obesity-linked cancer research.

## 1. Introduction

**Figure 1:**
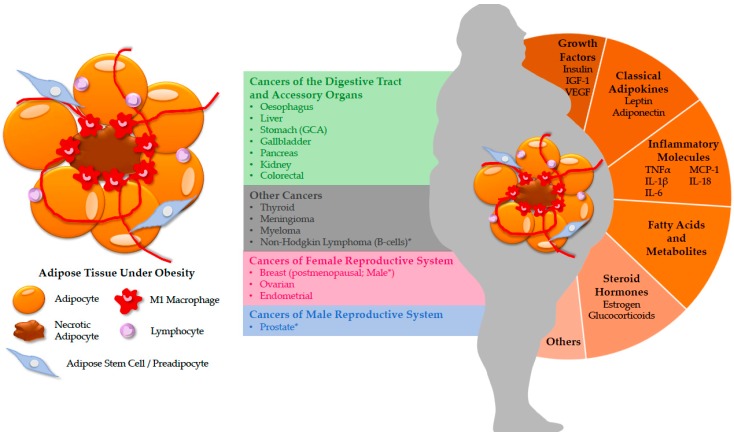
Obesity has been linked to more than 13 types of cancers in the body. The white adipose tissue (WAT) is home to diverse cell types: adipocytes, adipose stem cells, endothelial cells and many resident and infiltrating immune cells. During obesity, WAT releases a plethora of molecules with autocrine, paracrine and endocrine functions, including growth factors, adipokines, pro-inflammatory molecules, fatty acids (FA) and lipid metabolites and many others, which create a favorable condition for cancer to develop. Obesity has been shown to increase the risk of 13 cancer types and 3 others with limited evidence (*) [5]. The link(s) between obesity and its complications to multiple cancer types observed in humans are currently unknown. GCA: gastric cardia adenocarcinoma; IGF: insulin-like growth factor; VEGF: vascular endothelial growth factor; IL: interleukin. TNF: tumor necrosis factor; MCP: monocyte chemoattractant protein.

Obesity, in simple terms, is an increase in the white adipose tissue (WAT) of the body. Over the years, layers of complexity have been added to obesity studies by incorporating location and functional consequences of different adipose depots, influence from heterogeneous cell populations and secreted molecules, and local and systemic immunometabolic disruptions. Today, we are in an era where overnutrition kills more than malnutrition [1,2]. What evolved to be an advantage in energy storage has become detrimental in the modern world. After obesity is diagnosed by a Body Mass Index (BMI) of ≥30 kg/m^2^, a hurricane of complications often ensues, including high blood sugar and cholesterol, insulin resistance, elevated blood pressure, cardiovascular complications, metabolic syndrome and increased risk of cancers [3,4]. Cancer, a seemingly unrelated manifestation, may not be the first to come to mind nor an immediate concern regarding obesity, but this demonstrated linkage cannot be overlooked. Recent data have linked obesity to more than 13 different cancer types in the body, including those in the digestive tract and accessory organs (esophagus, stomach, colon, liver, kidney, gallbladder, pancreas), the female reproductive system (ovary, endometrium, postmenopausal breast), the male reproductive system (prostate) and others (meningioma, thyroid, multiple myeloma, and non-Hodgkin lymphoma) [1,5,6]. Together, these cancers account for about 8% of all cancer cases in North America [1]. Available statistics also suggest that in many western countries, obesity may have surpassed smoking as the leading cause of preventable cancer deaths [7,8]. Common mechanisms such as ectopic fat accumulation, altered adipokine production, hormone fluctuations and meta-inflammation (defined as a low-grade chronic inflammatory state induced under metabolic dysregulations) have been proposed to either independently or synergistically increase cancer risks of all types. The majority of cancers develops and metastasizes in the vicinity of an adipose tissue-rich environment. Initially, local inflammation begins with adipocyte hypertrophy and increased cell necrosis, which attract resident macrophages and promote recruitment of circulating monocytes, as evidenced by crown-like structures (CLSs) in the obese WAT. Interactions between hypertrophied adipocytes and pro-inflammatory immune cells further exacerbate the inflamed microenvironment, leading to meta-inflammation and cellular transformation. In parallel, overproduction of insulin and insulin-like factors under a state of insulin resistance promotes the proliferation of all cells, including cancers. While most adipokines and growth factors released under obesity induce general pathogenesis, others like estrogens have a more targeted influence over the female reproductive system. However, it is puzzling to researchers how a similar set of abnormalities produced by obesity leads to different types of cancers (Figure 1)**,** and the underlying mechanisms that produced these diversified cancers remain elusive. 

Obesity-related cancer takes time to manifest; and precisely for this reason, solutions are needed for early intervention. Although there are strong evidence of a link between obesity and cancer, the mechanisms have not been elucidated due to limitations in teasing obesity apart from its associated abnormalities and a lack of suitable pre-clinical models to study the obesity-linked cancer development. A vast majority of data supporting the obesity-cancer link come from observational studies, and experimental studies have only emerged in certain cancer types. With these challenges ahead, solutions are urgently needed not only to reduce obesity but also to understand its relationship with each cancer type and prevent a serious burden on the health care system in the coming years.

In this mini-review, we will summarize the most recent studies that examined the links between obesity and several common cancer types. In addition, we will discuss the limitations associated with mechanistic studies and the emerging rodent models that may help us better understand obesity-linked cancers. A detailed literature search on PubMed and Google Scholar was done for original research articles since 2012, reviews, and meta-analysis using the following search combinations: adipocyte/adipose tissue/obesity plus the cancer type. The references of selected publications were further searched for relevant information.

## 2. Cancers in the Digestive Tract and Accessory Organs

Central adiposity, composed of both visceral (omental, mesenteric, retroperitoneal) and subcutaneous fat depots, surrounds the digestive tract and its accessory organs including the liver, pancreas, and the gallbladder. These organs participate in the initial energy intake and processing that eventually lead to the accumulation of WAT. Central obesity together with diet and microbiota takes the blame for cancers of the abdominal cavity [9,10]. These three factors are often interconnected as high fat diet (HFD) can directly induce obesity or shift gut microbial composition to enhanced energy harvest that have both immune and metabolic consequences [11,12]. For example, HFD containing saturated fat and trans-fat promotes metastatic prostate cancer [13]; and, obesity-induced gut bacterial metabolites promote perturbed senescence phenotype and pro-inflammatory and tumor-promoting secretome in liver cancer [14]. Their interactions invariably lead to tissue-specific inflammation, including gastritis, hepatitis, pancreatitis and inflammatory bowel diseases, all of which propel their respective cancer types. Furthermore, men are more than twice as likely to develop cancers near the abdomen as compared to premenopausal women [15]. This advantage then becomes insignificant as weight “shifts” from pear-shape to apple-shape in postmenopausal women [16]. The difference in cancer susceptibility between genders has been well documented in cancer epidemiology [17,18]. Central adiposity, thus, may be a potential driving factor for sex disparity observed in cancers of this category. Here, we focus on obesity-linked gastric, liver and pancreatic cancers.

### 2.1. Gastric Cancer

Gastric cancer (GC) is an old-age and male-predominant cancer with late-stage diagnosis [19]. A recent meta-analysis of 24 prospective studies have revealed a positive correlation between patients with obesity and gastric cardia adenocarcinoma (GCA), cancer of the upper stomach and the most prevalent GC [20]. Increased intra-abdominal pressure, gastroesophageal reflux and altered levels of adipokines and cytokines (leptin, TNFα, IL-6 and IL-17) from obesity have been proposed as potential GCA promoting factors and reviewed in depth [21,22]. However, reason(s) behind obesity predilection for promoting GCA as opposed to cancer of the lower stomach (non-GCA) have not been elucidated.

In vitro studies examined interactions between human stomach cancer cell lines of the MKN series (MKN-7, MKN-28, MKN-45) and gastric subserosal adipose tissue stromal cells (ASCs), and demonstrated that ASCs promoted growth and invasiveness of GC cells while decreased cancer apoptosis through COX-2 independent MAPK activation in co-culture experiments [23]. Another study found ASCs also promoted GC cell growth through the chemotactic SDF-1/CXCR4 axis [24]. Leptin is the satiety hormone that positively correlates with adipose tissue mass, which is highly elevated in the plasma of most obese patients. One study found that leptin promoted GC cell migration through the RhoA/ROCK pathway and enhanced expression of intercellular adhesion molecule-1 (ICAM-1) [25]. Other studies suggested that leptin induced GC cell proliferation through JAK2/STAT3 and ERK pathways [26], which occurred after EGFR transactivation [27]. Of note, both adipocytes and gastric mucosa produce leptin in response to food intake, it is thus difficult to discern the autocrine and paracrine effects of leptin from different sources [28]. Co-cultures of isolated omental adipocytes with GC cells increased oleic acid uptake and enhanced invasiveness of GC cells, demonstrating a preference for lipid-rich fuels [29]. Other elevated adipocytokines such as IL-1β, IL-6, IL-17, and MCP-1 promote tumorigenesis by stimulating the Src/PI3K, MAPK, STAT3, and PKC pathways, some of which overlap with activated GC pathways mentioned earlier [30]. One study also looked at the synergic effect of infection and obesity and found that *Helicobacter felis*-infected mice fed on HFD accelerated gastric carcinogenesis through elevated immune response induced by adipose-derived IL-6 and leptin, and heightened pro-survival gene expression in gastric tissue through STAT3 signaling [31]. However, reason(s) behind obesity predilection for promoting GCA as opposed to cancer of the lower stomach (non-GCA) have not been elucidated. Available data have only demonstrated association between obesity and GC metastasis, while no research has examined its role in GC development.

### 2.2. Liver Cancer

In the United States, liver cancer has tripled in the last four decades with extremely low 5-year survival rate [32]. While the highest incidence remains in less developed nations, liver cancer is expected to rise in developed countries parallel to the obesity epidemic [33]. In addition to excess alcohol intake and viral hepatitis infection, obesity has become another contributing risk factor to hepatocellular carcinoma (HCC), the most common primary liver cancer [34,35]. A recent case-control study stratified by birth cohort, race/ethnicity, and sex reaffirmed previous findings that there is a positive correlation between high BMI and increased liver cancer risks [36]. In fact, HCC is the cancer type most enhanced by obesity through its tie with non-alcoholic fatty liver disease (NAFLD), non-alcoholic steatohepatitis (NASH) and type 2 diabetes mellitus (T2DM) [37]. Typically, obesity induces ectopic fat deposition in the liver and an imbalance of fatty acid accumulation and oxidation, resulting in steatosis. Chronic steatosis is a precursor for NASH that causes irreversible fibrosis and cirrhosis, which play significant role in HCC occurrence. These pathological processes have complex interactions, and their relative contribution at various stages in the development of liver cancer remains to be determined.

In cell culture studies, hypertrophied adipocytes, free fatty acids (FFAs) and adipocytokines released from the adipose tissue have all been shown to increase liver pathology. Co-culture study between hepatocytes and adipose tissue explants found more pronounced cytotoxic response and insulin resistance in hepatocytes co-cultured with inguinal compared to epididymal depots, signifying depot-specific effect [38]. Several researches have demonstrated that FFA treatment induced lipotoxicity in cultured hepatocytes, such as enhanced expression of inflammatory markers (IL-1b, IL-6, IL-8, TNFα), increased reactive oxygen species and fibrogenic cytokine productions in a dose-dependent manner [39,40]. FFAs also reduced the activities of cytochromes P450 for drug metabolism and fibroblast growth factor 21 for glucose and lipid metabolism [41,42]. It is speculated that FFA-induced JNK activation together with hyperinsulinemia further enhances apoptosis of hepatocytes [43]. Besides adiponectin, most adipokines derived from the adipose tissue exert a negative effect on liver diseases [44,45]. Other less understood secretion such as the exosome-enclosed microRNA-27a has also been shown to promote liver cancer cell proliferation through FOXO1 downregulation and an increase in G1/S cell cycle transition [46]. In addition, the bidirectional communication allows exosomes from liver cancer cells to activate pro-inflammatory NF-κB signaling pathway and associated phospho-kinases (Akt, ERK1/2, and GSK3β) in adipocytes, contributing to a positive inflammatory feedback loop [47]. Another hepatic-derived protein, unconventional prefoldin RPB5 interactor (URI), regulated by the mTOR pathway, has proven crucial in the crosstalk between liver and WAT as its high expression under HFD induced both hepatic DNA damage and exacerbated adipose tissue lipolysis, immune infiltration and insulin resistance [44]. Still, more factors are being identified that keep fortifying the link between obesity and liver cancer.

### 2.3. Pancreatic Cancer

Pancreatic ductal adenocarcinoma (PDAC) is the major (~95%) exocrine pancreatic cancer and the most lethal form of all pancreatic cancers. Obesity and T2DM both have close association with PDAC [48]. Central adiposity and fatty infiltration in the pancreas parenchyma are risk factors for pancreatic precancerous lesions, and fatty infiltration is significantly higher in PDAC patients with high BMI [49]. Obesity also promotes chronic pancreatitis, another inducer of PDAC that increases its risk by tenfold [50,51]. Pancreatitis-induced tissue damage results in inflammatory response primarily mediated by macrophages and T cells during early stages of tumor development in PDAC models [52]. The mechanism by which inflammatory cells facilitate progression of pancreatic intraepithelial lesions into invasive PDAC tumors remains to be determined. In general, epidemiological studies do not report on the type of pancreatic cancers associated with obesity. Despite the relative prevalence of PDAC, it is arguable that obesity poses higher stress on endocrine β-cells in the pancreas. During obesity, there is an initial expansion in β-cell mass followed by defects in β-cell function resulting in insulin resistance. However, to the best of our knowledge, the link between obesity and malignancy in β-cells (insulinomas) has not been examined.

The crosstalk between adipocytes and PDAC cells has been evaluated in a number of studies [53,54]. Like many cancers in the abdominal cavity, PDAC preferentially metastasizes toward the omental adipose tissue. Based on Ingenuity Pathways Analysis, the human omental fat produces enriched extracellular molecules related to cellular growth, migration, invasion and chemoresistance, with which nine proteins (NGAL, FINC, ZA2G, PGS1, TIMP1, MSLN, IL-6, MMP8 and TSP1) have been reported to specifically promote pancreatic cancers [54]. In the same study, omental fat conditioned media treated-pancreatic cells and xenograft undergo profound cellular reprogramming, leading to a more aggressive tumor phenotype. Especially under nutrient-poor conditions, adipocytes were found to transfer increased glutamine to PDAC cells with the help of PDAC cell-induced glutaminase downregulation [55]. Okumura group found that genetically engineered mice with Pdx1, Kras or Trp53 mutations fed on HFD had significantly larger primary pancreatic tumors and higher distant organ metastasis; in vitro, FFAs from visceral adipose tissue-derived conditioned media also increased lipid droplets in the pancreatic cancer cells and enhanced cell invasiveness [56]. A recent study has emphasized that Kras mutation alone is not sufficiently oncogenic, and upstream stimuli are needed to amplify its activity in tumors [57]. In this context, pro-inflammatory and growth factors secreted under obesity provide the push for sustained Kras activation and its downstream NF-κB, COX2 and STAT3 signaling, which are corroborated by animal studies [58]. The impact of inflammatory milieu is supported by evidence that high intratumoral IL1-β and TNF-α were associated with poor response to neoadjuvant therapy, and high IL-8 and GM-CSF were predictive of tumor grade and size [59]. In particular, IL-1β facilitated a self-sustaining loop of crosstalk between adipocytes, tumor-associated neutrophils and fibrogenic pancreatic stellate cells that enhanced desmoplasia, limited vascular perfusion, impaired chemotherapeutics and accelerated tumor growth [60]. Interestingly, pancreas-associated adipose tissue contained more functional adipocytes and higher expressions of insulin receptor and adiponectin, which protected the pancreas against short-term lipotoxicity through its increased capacity for energy storage and reduced inflammation markers [61]. Still, animal models with early pancreatic tumor led to adipose tissue wasting and increased cancer survival, which are hypothesized to be driven by decreased pancreatic exocrine function [62].

## 3. Cancers in the Female Reproductive System

Obesity increases the risks of ovarian, endometrial and breast cancers [63]. Studies have shown that women with a high BMI have greater incidence of polycystic ovarian syndrome (PCOS), endometrial polyps and fibroids, all of which aggravate gynecological cancer occurrence [64,65,66]. PCOS is independently associated with insulin resistance and glucose intolerance, two conditions that are exacerbated by obesity [67]. It may also be an indirect link between obesity and increased risks of both endometrial and ovarian cancers in premenopausal women [68]. Most female dominant cancers have indisputable connections to the fluctuating estrogen levels throughout life. In postmenopausal women, estrogens are locally synthesized at peripheral sites, mainly the adipose tissue, and take over the loss of gonadal estrogens [69,70]. The ability of adipose tissue to convert androgens to estrogens through the action of aromatase contributes greatly to the estrogen-sensitive cancer types [71]. Moreover, obesity-linked meta-inflammation are found to be a major driver for reduced sex hormone-binding globulins [72], which may further increase the bioavailability of sex hormones for local action. Inheritable mutation of BRCA1 or BRCA2 gene has been associated with a lifetime risk of both ovarian and breast cancers. Ongoing study has reported that BRCA mutation carriers are more susceptible to DNA damage in the breast epithelial cells under an obese environment [73]. In this category, we focus on obesity-linked breast cancer for its high prevalence in women and for it being one of the best studied obesity-related cancers in experimental researches.

### Breast Cancer

Breast cancer is the most common cancer in women, accounting for approximately one quarter (26%) of all new cancer cases and >12% cancer-related deaths in women worldwide [74]. Multiple studies have found that obese women with breast cancers have consistently poorer prognosis, larger tumors and lymph node metastasis [75,76,77,78,79]. The most common form develops from the lobules and ducts of the mammary gland surrounded largely by mature adipocytes and progenitors. While the relationship between local breast adipose tissue to metabolic risks and breast cancers has been conflicting and in need of standardization [80,81], it is apparent that the estrogen-rich microenvironment breast adipose tissue resides in and contributes to (through aromatase) plays a role in breast tumorigenesis [82]. Systemically, there is a strong link between obesity and breast cancer in postmenopausal women with inconsistent findings in premenopausal women [83,84,85]. A study on the Chinese population further incorporated the effect of adipose depots in breast cancer risk, showing premenopausal women with higher subcutaneous fat were more likely to develop hormone-receptor-positive breast cancer and postmenopausal women with higher visceral fat were more likely to develop hormone-receptor-negative breast cancer [86]. The vast heterogeneity in breast cancer etiology still remains elusive. In addition, it is worth noting that male breast cancer is on the rise with speculated connection to obesity and gynecomastia, the benign enlargement of the male breasts. 

Increased production of estrogens promotes the development of breast cancer; together with progesterone, they are classified as the hormone-receptor positive subtypes. A study in postmenopausal women found that estradiol levels in breast adipose tissue were lower in breast cancer patients than in controls whereas serum concentrations did not differ, which pointed to the importance of tissue-specific estrogen regulation [87]. Animal and cell studies found that close proximity between adipocytes and breast cancer cells is required for tumor growth and their interaction depended on adipose tissue aromatase expression through leptin signaling [88]. Inflamed WAT has elevated pro-inflammatory mediators, enhanced aromatase expression and estrogen receptor-α (ER-α)-dependent gene expression [89]. Intriguingly, estradiol also promoted growth of estrogen receptor-negative cancer cells through the activation of ER-α in breast stroma, which prevented hypoxia and necrosis in grafted tumors and normalized tumor angiogenesis [90]. Despite the importance of estrogens, it has become evident that the estrogenic aspect of obesity alone cannot account for a large subgroup of obese women identified with ER-negative breast cancer [91]. In addition to estrogens, other molecules released from the adipose tissue such as insulin, IGF-I, inflammatory cytokines and adipokines also collectively promote breast cancer development and progression [92,93,94]. Analysis of triple-negative breast cancer models fed on HFD during metastatic progression showed metabolic perturbation in FA metabolism, olfactory transduction, mTOR signaling and autophagy [95]. Reciprocal metabolic interactions have been observed between adipocytes and breast cancer cells, which are reviewed previously [96,97,98,99]. Their crosstalk has been observed to change global gene expression pattern (upregulated pathways: TNF signaling, NF-κB signaling, senescence & autophagy and cytokines & inflammatory response; downregulated pathways: cell cycles) [100], interference with the anti-proliferative efficacy of tamoxifen [101], and antibody-dependent cytotoxicity of trastuzumab [102]. Whole-genome transcriptome microarrays and protein analyses revealed that obesity accelerated ER+ breast carcinogenesis and progression through increased PI3K/AKT/mTOR signaling and adipokine secretion [103]. When co-cultured with ASCs from obese models, breast cancer cells showed enhanced survival and radioresistance [104] in part through leptin signaling [105]. Production of paracrine cytokines from ASCs also stimulated breast cancer cell growth through upregulation of cancer cell calcium-binding protein, which promoted tumorigenesis [106]. CLSs, indicative of inflamed and necrotic adipocytes, were more frequently seen in breast adipose tissue of breast cancer patients and correlated with increased estrogen to androgen ratios in both breast adipose tissue and serum [107]. High CLS density in breast adipose tissue has now been shown to independently increase breast cancer risk [108]. Notably, the expression of fatty acid binding protein common to both adipocytes and macrophages (A-FABP) in tumor-associated macrophages facilitated breast cancer progression through IL-6/STAT3 signaling [109]. In particular, the prominent immune checkpoint molecule, programmed death-ligand 1 (PD-L1), was found to be markedly elevated in mature adipocytes, which may saturate anti-PD-L1 antibodies and prevent the intended function of T cell activation in immunotherapy [110]. In fact, current clinical success of cancer immunotherapy in breast cancers has been limited and could benefit from understanding of adipose-immune-tumor interactions [111].

## 4. Cancers in the Male Reproductive System

Low testosterone level in men correlates with increased central adiposity and reduced lean mass, energy imbalance and an overall impaired metabolism [112,113]. A decline in testosterone is attributed partly to a decrease in sex hormone-binding globulin associated with obesity and an increased aromatase conversion of estradiol in adipocytes [114,115]. Currently, the only male-specific cancer connected to obesity is prostate cancer. Despite controversies, low testosterone level is associated with a higher risk and worse outcomes for prostate cancer [116,117]. Several studies suggest an increased risk of aggressive prostate cancer in obese men, and possible links between obesity and prostate cancer have been reviewed in a dated publication [118]. In contrast, although obesity is shown to disrupt testicular morphology and spermatogenesis that cause infertility and induce chronic inflammation in the male reproductive system [119,120], no study has established a link between obesity and testicular cancer. For invasive penile cancer, obesity is included as a risk factor because of speculated and indirect mechanisms such as inflammation and obesity-induced buried penis [121,122,123]. For these andrological cancers, adipose tissue does not have direct contact and appears to facilitate primarily through ancillary inflammation, though further investigation is needed. In this category, we will survey the most recent findings on obesity-linked prostate cancer.

### Prostate Cancer

The most common prostate cancer (PC) termed acinar adenocarcinoma develops from glandular cells surrounding the prostate. Current treatment for advanced PC is based on androgen deprivation to which tumor cells initially respond to, but eventually they become castrate-resistant by acquiring changes such as androgen-receptor overexpression [124]. Men with metastatic castrate-resistant PC have a median survival of less than 2 years and no standard therapeutic modality [125]. While only limited evidence has found a link between obesity and PC, their overlapping trend among older men suggests the likelihood that PC will continue to grow in light of the obesity pandemic. The pathophysiological and biological mechanisms underlying these associations are only beginning to be understood [126].

Periprostatic adipose tissue inflammation is commonly observed in PC patients [127]. Gene expression signature of periprostatic WAT in obese and overweight patients exhibited hypercellularity and reduced immunosurveillance [128]. Chemokine C-C motif ligand 7 (CCL7) produced in periprostatic WAT was found to stimulate the migration of chemokine receptor type 3-expressing tumor cells [129]. Multiple reviews proposed that obesity-induced inflammation is a major culprit in PC [130,131]. Within adipocytes, loss of p62 (a ubiquitin-binding protein in autophagy) shut-downed mTOC1-mediated FA metabolism, which increased FA availability for PC cells [132]. Interestingly, adipocyte specific p62-deficient mice developed obesity independent of diet and displayed enhanced synthesis/secretion of posteopontin from adipocytes and carnitine palmitoyltransferase Ia from tumor cells, both of which are predictors of poor PC prognosis [132]. PC exosomes also assisted adipocyte lipolysis through the p38 and ERK pathways and hormone-sensitive lipase activation, which were proposed as mechanisms to enhance pancreatic local invasiveness and metastasis [56,133]. Paracrine molecules from adipocytes were found to stimulate the non-canonical WNT signaling pathway in PC cells, leading to nuclear translocation of genes regulating epithelial-to-mesenchymal transition and cancer aggressiveness [134]. In addition, the periprostatic WAT also showed increased matrix metalloproteinase activity, which is essential in extracellular matrix remodeling that promotes PC cell proliferation and motility [135]. On the other hand, obesity is associated with decreased concentration of testosterone, which reduces the risk of hormone-sensitive PC but paradoxically increases the risk of aggressive PC [136]. Androgens can act both as a stimulator of lipolysis and regulator of several inflammatory genes [137,138]. The role of androgens in obesity and PC has been well established and independently studied, but their potential interplay has yet to be determined. 

## 5. Current Approaches to Study Obesity-Linked Cancers and Their Limitations

**Figure 2:**
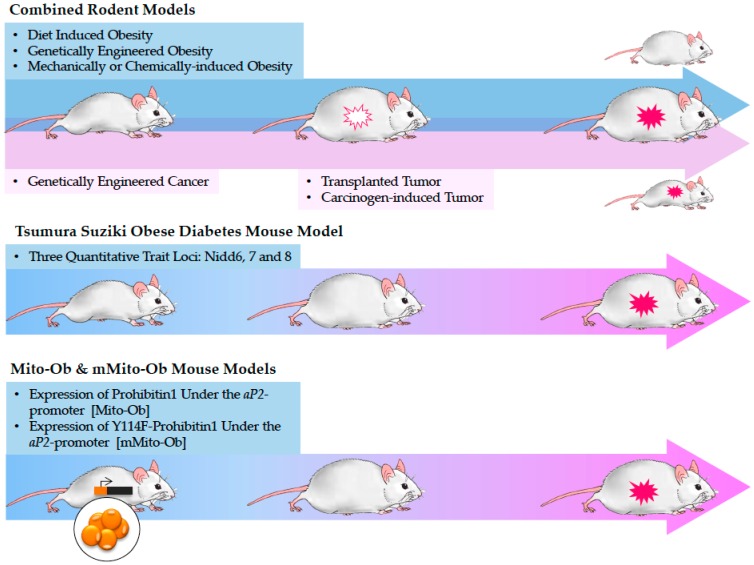
The development of obesity-linked tumors in combined rodent models, Tsumura Suzuki Obese Diabetes (TSOD) and Mito-Ob/mMito-Ob mouse models. Tsumura Suziki Obese Diabetes (TSOD) and Mito-Ob/mMito-Ob mouse models. The combined rodent models develop obesity and cancer through independent manipulations in genetics, diet, and inducible techniques [139,140]. They are excellent models to study cancer progression under an obese environment. Since the two morbidities can develop independent of each other, these models do not address whether obesity initiates tumorigenesis spontaneously. Both the male TSOD and Mito-Ob/mMito-Ob mouse models develop tumors spontaneously from genetic manipulations that led to an obese and diabetic phenotype, which may better explain the inducible cancer risks from obesity. For this mini-review, only rodent models are discussed, as they are the most commonly used animals. We acknowledge that other models exist and contribute significantly to obesity/cancer research but remain outside the scope of this mini-review. Nidd: non-insulin-dependent diabetes; aP2: adipocyte protein 2.

The most difficult question in obesity-linked cancer research is to identify whether obesity independently increases the chance of cancer without its associated conditions. In vitro studies often examine interaction between adipocytes and cancer cells of interest using co-culture experiment or treatment with conditioned media from adipocytes. Emerging multicellular 3D models also more closely mimic the tumor microenvironment and its behavior. Direct cell-cell communication is vital in cancer proliferation and metastasis, but systemic interactions and hormone regulations from the adipose tissue require analysis in whole organism. In vivo approach combines oncogenic model [139] with obese model [140] in order to replicate obesity-linked cancer development and progression in humans (Figure 2). These combined models have provided valuable information on the effect of obesity on tumor progression as exemplified by some high impact studies from the previous sections. However, they fell short in a number of ways in elucidating the link between obesity and the onset of cancer. First, oncogenic models develop tumors regardless of an obese background. Most combined models observed an exacerbated tumor phenotype under an obese environment signifying aggressive progression of existing tumor but could not address whether obesity initiates tumorigenesis spontaneously. Second, combine models introduce confounding effects of diet, leptin deficiency and carcinogens in addition to obesity on tumor development/progression. For example, in DIO, the effect of HFD itself may be an independent risk factor for cancer. Other obese models such as the ob/ob and db/db, have a defect in leptin or the leptin receptor, which prevent leptin regulation on obesity-associated signaling pathways in cancer. Under an obese state in breast cancer, leptin interacts with molecular effectors such has estrogen, insulin, IGF-1 and inflammatory cytokines, which impacts various stages of breast cancer from initiation to metastatic progression [141]. Transplanted and carcinogen-induced tumors occur after obesity is established, and again do not replicate cancers that develop from a progressive and systemic change due to obesity. Although statistical techniques to adjust for confounding variables can improve epidemiological comparisons, there is still a lack of translational model to study the relationship between obesity, insulin resistance and tumor development, as well as the relative contribution of obesity-associated abnormalities to cancers. To the best of our knowledge, none of the combination model that pairs obesity to cancer develops obesity- or diabetes-linked cancer spontaneously and sequentially like those found in humans.

### 5.1. Tsumura Suziki Obese Diabetes (TSOD) Mouse Model

The male TSOD mouse model has been reported to spontaneously develop obesity-associated diabetes, NASH, and HCC by 10 months without additional gene manipulation [142,143]. The phenotypes of this inbred ddY strain have been attributed to three quantitative trait loci involved in blood glucose level and body weight: non-insulin-dependent diabetes (Nidd) 4 on mouse chromosome 11, Nidd5 on mouse chromosome 2, and Nidd6 on mouse chromosome 1 [144]. However, the gene(s) responsible for the observed hepatic tumorigenesis is currently unknown. At a young age (about 5 weeks), TSOD mice start gaining weight in both the visceral and subcutaneous adipose tissues, and develop high plasma triglyceride and cholesterol levels accompanied by oxidative stress marker, but not hyperglycemia [142,145]. Adipocyte hypertrophy, pro-inflammatory macrophage-induced CLSs and CD8-positive lymphoid aggregation were observed at 4 months, together with ballooning and microvesicular steatosis in the liver [146]. Multi-organ changes at advanced age were noticed, including severe hypertrophy of pancreatic islets due to B cell infiltration, thickening of the basement membrane in glomeruli, and motor and sensory neuropathies, closely mimicking the diabetic complications [147]. In TSOD mice that developed HCC, a high frequency of glutamine synthase expression and overloading of intrahepatic bile acids were observed, which resemble HCC in humans [143,148,149]. Of note, TSOD mice develop obesity due to disrupted control of hypothalamic-mediated hyperphagia that persist throughout diabetes and HCC, making it difficult to discern the effect of obesity on HCC from coexisting diet and diabetes [150]. Furthermore, TSOD mice also have both low sympathetic and adrenomedullary activities with high adrenocortical activity starting 4 months of age, which affect central energy regulation and hormone secretion and add extra linkages to HCC [151]. Since its creation, the TSOD model was only established and characterized in males; selective breeding in obese females also led to significant weight increase but showed no sign of urinary glucose [152]. This phenomenon is not unique to TSOD mice and has been reported in other polygenic mouse models [153]. It would be thus interesting to follow up on the male sex bias in metabolic syndrome severity. Nonetheless, the TSOD mouse model represents a promising tool to study the obesity-T2DM-NASH-HCC axis.

### 5.2. Mito-Ob and mMito-Ob Mouse Models

Irrespective of etiology, obesity is characterized by an increase in adipose tissue mass that requires corresponding changes in adipocytes, the structural and functional units of adipose tissue. Through adipocyte remodeling, a transgenic mouse model, Mito-Ob, developed obesity through an upregulation of prohibitin (PHB) under the adipocyte protein 2 (*aP2*, also known as *Fabp4*) promoter. PHB is a highly conserved and pleiotropic protein found in multiple subcellular compartments such as the mitochondria, cytosol and nucleus. Its predominant role in many cells has been in maintaining mitochondrial homeostasis and biogenesis, though it is also intricately involved in insulin sensitivity, proliferation and immune regulation in part through phosphorylation at tyrosine (Y)-114 [154,155]. Longitudinal studies on these mice uncovered obesity-related metabolic disruptions and a spontaneous development of HCC specifically in males [156,157]. A mutant (m)Mito-Ob mouse model that overexpresses a phospho-mutant PHB (Y114F) under the aP2 promoter also developed obesity and diabetic symptoms, but instead manifested lymphoproliferative disease (lymph node tumors) or adult onset autoimmune diabetes when fed on HFD [158]. It appears that mutation at a phosphorylation site in the adipose tissue-specific PHB gene altered the course of cancer development and produced multiple cancer types under the same obese background. Of note, since aP2 gene is expressed in both adipocytes and monocytic immune cells (macrophages and dendritic cells), PHB expression may be altered in both cell types. The distinct cancer types in Mito-Ob and mMito-Ob mice imply that PHB Y114F mutation may have activated an immune dysregulation mediated by enhanced PI3K-Akt signaling in monocytic cells, which increased the likelihood of immune-base complications such as the observed lymph node tumor and autoimmune diabetes [155]. These results suggest tissue- and compartment-specific functions of PHB, which are known in the literature [159]. It is speculated that PHB over-expression in adipocytes was sufficient to induce obesity, while Y11F mutant PHB over-expression preferentially affected monocytic cells. Research is currently underway to extrapolate the puzzling difference in mechanisms between the metabolic and immune functions of PHB. Both Mito-Ob and mMito-Ob mice have shown spontaneous development of cancers from an obesity background independent of diet and carcinogens, which may serve as a model to study obesity-cancer linkage. Furthermore, the metabolic dysregulations in female Mito-Ob mice appeared after ovariectomy, suggesting involvement of estrogens in regulating PHB [158]. These findings are consistent with recent WHO report proposing possible roles of sex steroids and chronic inflammation in obesity-linked cancers [5]. Thus, research going forward should be mindful of sex difference in the context of obesity and the development of all obesity-linked cancers. 

## 6. Conclusions and Future Perspective

This review highlights the current knowledge on obesity-linked cancers, their speculated mechanisms and rodent models used to study the link. Now, 13 cancers have been confirmed to have an increased risk in obesity. It is likely that more cancer types will be added to the list given the systemic influence obesity and its complications have. To top it off, childhood and infant obesity are also on the rise, both of which are strong predictors of adult obesity [160,161,162]. While no evidence has linked obesity to any pediatric cancers, recent report has suggested that obesity accelerate cancer occurrence at a younger age [163]. Survivors of childhood cancer are also at increased risks for obesity, T2DM and cancers in adulthood [164]. In many cases, obesity-related abnormalities are the main facilitators of cancer development, not obesity per se. For example, the A-Zip/F1 mouse model, which has virtually no WAT, showed high serum glucose, insulin, IGF-1 and pro-inflammatory cytokine levels, and accelerated tumor formation [165]. Human studies have also identified a subset of up to 25% of obese individuals that are metabolically healthy, but little is known about their long-term metabolic outcome [166]. On the contrary, the metabolic syndrome has also been observed in lean individuals, pointing to the likelihood that obesity itself may not be sufficient for disrupted metabolism [167]. It would be interesting to extrapolate the differences between metabolically healthy obese and metabolically unhealthy lean individuals. In fact, the role of obesity-associated complications in cancer development has been strongly supported by the use of Metformin (an antidiabetic, glucose-lowering drug) and Aspirin (anti-inflammatory drug) in cancer prevention and treatment [168,169]. Obesity and related metabolic changes can be reversed through interventions, but whether obesity-induced cancer can be reversed is unknown. It raises the concern that losing weight in chronically obese patients may not be sufficient to reduce the risk of cancer development by significant amount. While obesity has been confirmed as an independent risk factor for cancers, it is extremely difficult to discern between obesity and its comorbidities in driving cancer. In this regard, monitoring the progression and metabolic profile at different stages of obesity (as proposed by the Edmonton Obesity Staging System [170]) may be useful in assessing obesity-related risks and prioritizing treatment. In any obesity-linked cancer, it is likely that dormant mutations are activated locally by inflamed adipose tissue or by secreted endocrine molecules at a distance. The proximity effect of obesity heavily depends on body adipose tissue depot distribution, composition and physiology to which tumorigenesis requires. The synergic effect of other carcinogenic factors such as diet in gastric cancer, alcohol in liver cancer, menopause in gynecological cancers and infections in general can further promote cancer of a particular type. Additionally, epigenetic modifications from obesity that are oncogenic cannot be ruled out. Studies on genetically identical mice and monozygotic twins found that changes in a chromatin-interacting protein, Trim28, resulted in an epigenetic binary switch (produced by a group of imprinted genes) of either a lean or obese phenotype [171]. Another study directly linked epigenetic changes during obesity to histone modifications and DNA methylation in the colonic epithelium, which preceded a tumor-prone gene signature that is reversible through weight loss [172,173]. Much of these findings still require rigorous follow-ups. Research carrying forward should build upon available pre-clinical models to examine the relationship between excess adiposity and the development and progression of individual cancer, while bearing in mind the need to distinguish potential comorbidities and advance our understanding for preventative and therapeutic strategies.
